# A *Gallus gallus* Model for Determining Infectivity of Zoonotic *Campylobacter*

**DOI:** 10.3389/fmicb.2019.02292

**Published:** 2019-10-22

**Authors:** Dennis Lye, Ian Struewing, Theresa M. Gruber, Kevin Oshima, Eric N. Villegas, Jingrang Lu

**Affiliations:** ^1^Office of Research and Development, USEPA, Cincinnati, OH, United States; ^2^Pegasus Technical Services, Inc., Cincinnati, OH, United States

**Keywords:** *Campylobacter*, colonization, chick model, infectivity, gull, avian

## Abstract

To better understand public health implications of waterfowl as reservoirs for zoonotic sources of *Campylobacter* in recreational waters, we developed a *Gallus gallus* (chick) model of infection to assess the pathogenicity of environmental isolates of *Campylobacter*. This method involved exposure of 1-day-old chicks through ingestion of water, the natural route of infection. Viable *Campylobacter* from laboratory-infected animals were monitored by using a modified non-invasive sampling of fresh chick excreta followed by a passive polycarbonate-filter migration culture assay. The method was used to evaluate the infectivities of three laboratory strains of *Campylobacter* spp. (*Campylobacter coli, Campylobacter jejuni*, and *Campylobacter lari*), three clinical isolates of *C. jejuni*, and four environmental *Campylobacter* spp. isolated from California gulls (*Larus californicus*). The results revealed that chicks were successfully infected with all laboratory and clinical isolates of *Campylobacter* spp. through ingestion of *Campylobacter*-spiked water, with infection rates ranging from <10 to >90% in a dose-dependent manner. More importantly, exposure of chicks with *Campylobacter* spp. isolated from *Gallus gallus* excreta also resulted in successful establishment of infection (≤90%). Each monitored *Campylobacter* spp. contained ≥7.5 × 10^4^ CFU⋅g^–1^ of feces 7 days post-exposure. These results suggest that a *G. gallus* model can be used to assess infectivity of *Campylobacter* isolates, including gull and human clinical isolates. Use of an avian animal model can be applied to assess the importance of birds, such as the *G. gallus*, as potential contributors of waterborne-associated outbreaks of campylobacteriosis.

## Introduction

*Campylobacter* spp. bacteria are a major cause of zoonotic human enteric infections commonly transmitted by ingestion of contaminated food or water (Pitkänen, 2013). These bacteria, which are commensal organisms within the gastrointestinal tract of various animals, including birds, have also been isolated from contaminated fresh and marine recreational beach sites (Savill et al., 2001; Stoddard et al., 2005). Recent studies have suggested that *Campylobacter* spp. present in a water source often reflect the source(s) and/or type of fecal pollution at that site. For example, *Campylobacter jejuni* is most associated with sewage discharges, whereas *Campylobacter coli* and *Campylobacter lari* are associated more with agricultural runoff and/or the presence of abundant waterfowl fecal contamination (Pitkänen, 2013). Because *Campylobacter* spp. require specific fastidious environmental parameters for growth, they are unable to multiply and persist in most surface waters (Obiri-Danso et al., 2001); thus, detection of culturable *Campylobacter* spp. in surface waters is usually an indication of recent fecal contamination.

Amid increasing public concerns about transmission of enteric disease from waters harboring large bird populations, fecal releases from wild birds have been reported to have a significant role in water quality impairment of recreational waters (Lévesque et al., 2000). Additionally, seagulls and ducks have been reported to be major contributors of *Salmonella* and *Campylobacter* bacteria via release and dispersal of their feces in recreational waters (Kapperud and Rosef, 1983; Quessy and Messier, 1992). The extent of colonization and persistence of various *Campylobacter* spp. within wild birds remains largely undetermined. Ramos et al. (2010) reported a direct relationship between *Campylobacter* infection of fledgling gull chicks with exposure/consumption diets within human-altered environments (particularly related to garbage and sewage). The colonized gulls showed no adverse health impacts, which could lead to the potential dispersal of *Campylobacter* over large geographical areas (Bingham-Ramos and Hendrixson, 2008).

Understanding the role of waterfowl as reservoirs for zoonotic *Campylobacter* spp. in recreational waters has important public health implications. The animal models (piglet, mouse, and rabbit) to assess the infectivity and pathogenicity of different strains of *Campylobacter* have been used (Field et al., 1981; Babakhani et al., 1993; Hodgson et al., 1998; Stahl and Vallance, 2015; Giallourou et al., 2018; Hartley-Tassell et al., 2018). Considering that *Campylobacter* spp., especially *C. jejuni*, have evolved to preferentially colonize the avian gut, the chick model was developed and is the more relevant animal model for investigating bacterial colonization factors (Newell, 2001; Müller et al., 2006; Manes-Lazaro et al., 2017; Sweeney et al., 2017; Salaheen et al., 2018). Although animal models have also been shown to be useful for investigating *Campylobacter* virulence factors involved during infection, these approaches have inherent limitations, specifically the use of invasive procedures and surgical intervention and/or atypical administration of bacterial inoculums (via oral inoculation or gavage) that do not mimic natural infections with *Campylobacter* (Ringoir et al., 2007; Stern, 2008). Invasive sampling procedures, such as terminal surgical tissue (Stern, 2008; Clavijo and Flórez, 2017) or cecal swab sampling (Cawthraw et al., 1996; Ringoir et al., 2007), have also been used. Together, these approaches may alter animal behavior and susceptibility to *Campylobacter* infection and potentially affect accurate assessment of susceptibility to and persistence of *Campylobacter* infections.

Detection and isolation of *Campylobacter* spp. from the environment are laborious and often based on enrichment procedures, selective media, and antibiotic resistance. Moreover, many environmental *Campylobacter* isolates are sensitive to antibiotics, which makes it difficult to use selective culture-based detection (Steele and McDermott, 1984). Nevertheless, an improved method has been reported that uses a passive-filtration plating technique for isolation of *Campylobacter* spp. from environmental waters and animal samples; the method uses size exclusion filters to select for “smaller” highly motile *Campylobacter* from most other “larger” less motile bacteria that may be found in environmental samples (Steele and McDermott, 1984; Jokinen et al., 2012). In a subsequent study, the use of polycarbonate filters gave increased recovery of *Campylobacter* spp. from stool samples when using the same passive-filtration plating procedure (Nielsen et al., 2013). This passive-filtration technique can be valuable for determining the viability of environmental *Campylobacter* isolates. Thus, this filter method should be a suitable approach for this study.

The aim of this study was twofold: (1) develop a non-invasive chick model of infection that mimics the natural route of infection to assess the infectivity of environmental *Campylobacter* isolates, and (2) use the passive-filtration plating technique to assess the bacterial burden in animals following infection, including the persistence of various *Campylobacter* spp. in a natural host. More broadly, since the prevalence and zoonotic potential of *Campylobacter* spp. found in wild avian species and recreational waters remains poorly understood (Weis et al., 2014, 2016) the techniques described herein will be useful toward developing more accurate risk assessment models of waterfowl-derived *Campylobacter* spp. human infections in a recreational water exposure scenario.

## Materials and Methods

### Animals

Specific pathogen-free fertilized chicken layer eggs (*Gallus gallus*) were obtained from Charles River Laboratories (North Franklin, CT, United States) and were incubated upon receipt (37–38°C, at 45–55% relative humidity) for 21 days, with occasional rotation. On day 18, the eggs were placed on hatching trays at a temperature range of 31–32°C and a relative humidity of 60–65% and allowed to hatch with no additional rotation. Once the chicks hatched, were fully dry, and were able to walk, they were transferred from the hatchery and placed in an individually ventilated cage (IVC) system with the temperature maintained between 32 and 38°C throughout the experiment. The chicks were then randomly assigned to one of the three dose groups (A, B, or C; Supplementary Table S1) per *Campylobacter* isolate (with 17–23 chicks per group, *n* = 630). There is no “intermixing” between or among the strains with this system. Once randomly assigned to a dose group and placed into the IVC system, they were given the infected water. Both the chamber and cage system were sterilized prior to placing the eggs and chicks. All animal experiments were approved by the US Environmental Protection Agency (USEPA) Animal Facility Oversight of Institutional Animal Care and Use Committee.

### Bacterial Isolates

Ten *Campylobacter* species/isolates were analyzed in this study: three laboratory, three clinical, and four environmental isolates. The laboratory isolates were *C. jejuni* (ATCC 29428), *C. coli* (ATCC 33559), and *C. lari* (ATCC 35221) (American Tissue Culture Collection, Manassas, VA, United States). The clinical isolates were cultured specimens taken from human fecal samples of diagnosed *C. jejuni* infections (kindly provided by a local hospital doctor). The environmental isolates (58BB: *C. lari*; 63A: *C. jejuni*; 64BB: *C. lari*; 70BB: *Campylobacter volucre*) (Supplementary Table S2) were obtained from California gull (*Larus californicus*) fecal samples collected from Southern California Hobie Beach as previously described (Lu et al., 2011). All isolates were grown and maintained on 5% sheep blood agar plates, as described below. Their physiological and biochemical characteristics were tested and summarized in Supplementary Table S3. For long-term storage, frozen (−80°C) glycerol stocks were made for each clone used in this study, as previously described (Han et al., 1995).

### Inoculum Preparation of *Campylobacter* spp.

*Campylobacter* spp. were cultured by using sheep blood agar plates (SBAP) containing 5% sheep blood (VWR International Inc., Radnor, PA, United States) incubated for 48–72 h at 37°C, which is considered as optimal growth temperature (Hsieh et al., 2018), in microaerophilic chambers (Mitsubishi AnaeroPack System, Fisher Scientific, Hanover Park, IL, United States). Single *Campylobacter* colonies were transferred by swab onto four fresh SBAPs and incubated for 48 h at 37°C in a microaerophilic chamber until a full bacterial lawn on each plate was achieved. Lawns from these plates were then harvested by flooding each plate with 20.0 mL of sterile water and then pooled and diluted until an optical density_640_ = 0.14 was achieved. This initial suspension of *Campylobacter* was then diluted 10-fold with sterile water, which typically resulted in titers of about 1 × 10^7^ CFU mL^–1^. To make sure that animals would show some infection (colonization) under the lowest dose, two additional 10-fold serial dilutions were prepared in sterile water to give a total of three different inoculum doses of culturable *Campylobacter* used throughout the study (Supplementary Table S1). The volumes prepared for each inoculum dose were sufficient to provide 100 mL for each chick for 24 h use. Since *Campylobacter* spp. have been shown to be sensitive to light and temperature (Obiri-Danso et al., 2001) and can become non-culturable within 30 min of exposure to artificial light according to our test (data not shown), fresh *Campylobacter* suspensions were made for each experiment and protected from light in 15-mL conical tubes wrapped with aluminum foil and kept on ice (or refrigerated) until used.

### Infection Through Natural Ingestion of *Campylobacter*-Containing Drinking Water

Fifty milliliters of freshly prepared *Campylobacter* suspensions, as described above, were dispensed into sterile isolator containers (Bio Serv, Flemington, NJ, United States) and placed into individual chick cages. An initial 50-mL inoculum solution was placed in the cages for 8 h and then replaced with a new 50-mL inoculum solution of the same suspension dilution for an additional 16 h before replacing with sterile drinking water. The viability of inoculum after 8 h in the cage only had slight decrease. The total volume of inoculum solution ingested by each chick was monitored and calculated for the entire 24-h exposure period. Total bacterial numbers ingested were calculated on the basis of the volume ingested and densities of *Campylobacter* suspension (Supplementary Table S1). Uninfected control chicks (*n* = 22) just received the same sterile water and were monitored in a manner similar to that for the infected group. Six hundred and thirty 1-day-old chicks were exposed to three different concentrations of laboratory, gull, and human clinical isolates of *Campylobacter* spp. and monitored over a 7-day period.

### Assessing Bacterial Burden in Chick Feces by Using the Passive-Filtration Culture Method

Fresh fecal materials collected at two time points (2 and 7 days) were assayed by using a modified passive-filtration culture method (Steele and McDermott, 1984; Jokinen et al., 2012). Briefly, individual chicks were placed in a cage containing sterile paper bottoms and allowed to defecate. Within 10–15 min, most chicks had defecated onto the sterile paper, and samples were taken by swiping a sterile swab through the freshly deposited feces. Each fecal swab sample with an average of 390 mg was placed into a tube containing 3.0 mL of sterile water (held on ice) and agitated to dislodge and disperse the fecal matter. After agitation, 0.1 mL, which contained an average of 13 mg of the fecal specimens, was plated directly onto a SBAP plate (100 mm in diameter) with two 47-mm diameter polycarbonate filters of 0.6-μm pore size (GE Water & Process Technologies, Addison, IL, United States) placed side by side and on top. The filter pore and diameter size provided optimal results for culturing *Campylobacter* spp. for this study (data not shown). Samples were then spread gently and evenly across the two filters. Finally, the SBAPs were incubated at 37°C for 45 min (no microaerophilic chamber). After 45 min, the filters were removed from the surface by using a sterile forceps, and the SBAPs (without filters) were placed in a microaerophilic chamber and incubated at 37°C. *Campylobacter* colonies were enumerated after 48 h of incubation. On the basis of the processed 14–27 replicates with no-dilution, the Most Probable Number (MPN) calculation program (version 5) (Jarvis et al., 2010) was used to perform MPN analyses. A number of colonies were randomly tested and confirmed for the targeted strains using qPCR methods (Supplementary Table S4) mentioned in previous study (Lu et al., 2013).

### Data Analysis

To check for normality of the data, the Shapiro–Wilk test was performed. As the data were not normally distributed, the non-parametric Kruskal–Wallis One Way Analysis of Variance on Ranks was used to determine if the differences in the median values among treatment groups were greater than would be expected by chance. If a statistically significant difference (*p* ≤ 0.05) was found, Dunn’s test of multiple comparisons was conducted to isolate the group or groups that differed from the others. All statistical tests among different strains, different doses, were done via SigmaPlot 13.0.

## Results

### Comparison Using Spread Plate vs. Passive-Filter Culture Assays of *Campylobacter* spp. Recoveries

We compared the traditional spread plate with the passive-filter technique for culturing of three laboratory strains (*C. jejuni, C. coli*, and *C. lari*), three clinical strains (C1–3), and four gull isolated strains of *Campylobacter* species (Table 1). The results revealed differences in total colony counts between the spread plate vs. passive-filter culture techniques. Total colony counts in cultures grown by using the spread plating were higher (2 × 10^6^ − 1 × 10^8^CFU mL^–1^) than those grown by using passive-filter plating (2 × 10^5^ − 5 × 10^6^ CFU mL^–1^). The colony ratio detected between the two plating types ranged from 6 to 30 (mean = 16). In addition, the passive-filter technique proved to be less labor intensive and did not require additional sample dilution to avoid overcrowding of the plate (data not shown). On the basis of the added technical advantages and a built-in enrichment step provided by the passive-filter culture technique in this study, as well as on reports by Nielsen et al. (2013), all subsequent experiments described below used this bacterial culture procedure.

**TABLE 1:** Comparisons of spread plating vs. passive filter platings to assess total viability of various laboratory, clinical, and environmental *Campylobacter* strains (CFU mL^–1^).

**Isolate**	**Titer from spread plating^a^**	**Titer from passive -filter plating^b^**	**Ratio of spread count vs. filter count cells^c^**
**Laboratory**			
*C. coli*	3 × 10^7^	1 × 10^6^	30:1
*C. jejuni*	4 × 10^7^	5 × 10^6^	8:1
*C. lari*	1 × 10^7^	1 × 10^6^	10:1
**Environmental**			
64BB	3 × 10^7^	2 × 10^6^	15:1
58BB	8 × 10^6^	6 × 10^5^	14:1
70BB	3 × 10^7^	5 × 10^6^	6:1
63A	5 × 10^7^	2 × 10^6^	25:1
**Clinical**			
C1	2 × 10^6^	2 × 10^5^	10:1
C2	1 × 10^8^	5 × 10^6^	20:1
C3	1 × 10^7^	4 × 10^5^	25:1

*^*a*^Titers calculated from 0.1 mL of bacterial suspension dilutions plated directly onto BAP plates; ^*b*^Titers calculated from 0.1 mL of the same bacterial suspension dilutions plated onto BAP-containing polycarbonate filters as described in section “Materials and Methods”; ^*c*^Ratio of culturable cells calculated from spread plates vs. passive-filter plates.*

### Infection of Chicks With *Campylobacter* Laboratory Isolates

**FIGURE 1:**
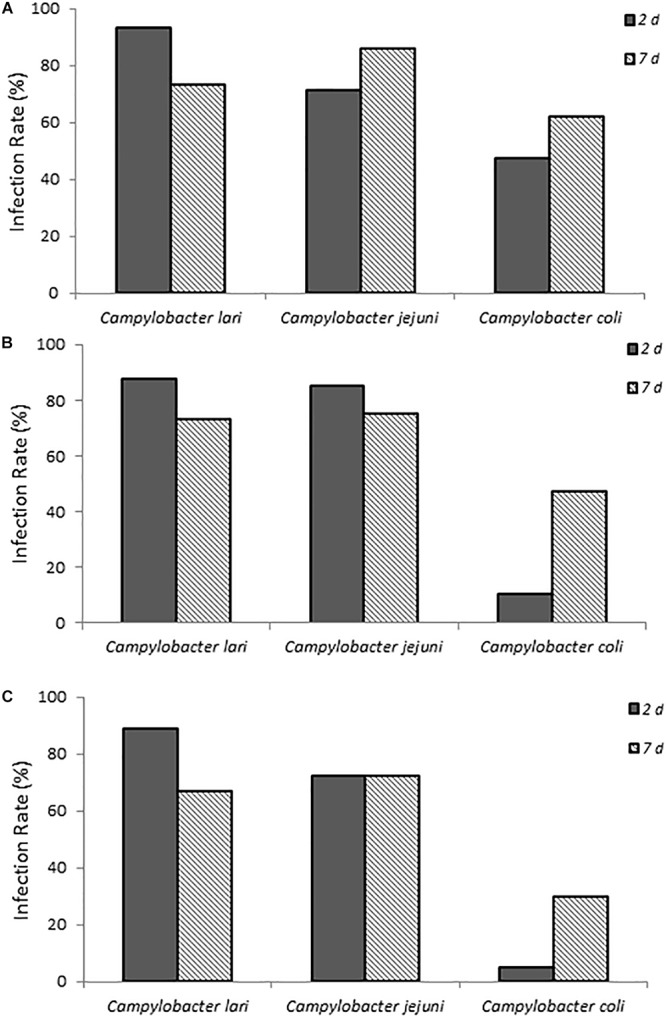
Infection rates of laboratory isolates of *Campylobacter* spp. **(A–C)** show chicks infected with dose A, B, and C *Campylobacter* spp., respectively. Percent infected was determined by dividing the number of infected chicks by the total number of chicks inoculated with the various *Campylobacter* species. Chicks were considered infected if fecal samples collected at the various times were positive for *Campylobacter* spp. as determined by using the passive-filter plating described in section “Materials and Methods.”

The ability of three laboratory isolates of *C. coli, C. jejuni*, and *C. lari* to successfully infect 1-day-old chicks was evaluated. As shown in Figure 1, chicks exposed to various concentrations of *Campylobacter* spp. (Supplementary Table S1) via a natural ingestion route, as described above, were successfully infected. By contrast, uninfected control animals remained negative for *Campylobacter*. The three different laboratory strains showed significantly different patterns of infection rates (*p* < 0.05, compared at 3-doses and at 2-day time points), which were detected up to 7 days post-infection (Figure 1). *C. jejuni* had the most consistent colonization rates ranging from 71.4 to 85.7% of animals that were positive at both time points evaluated and at all three doses. *C. lari* had the highest infection rates at all three doses at 2 days post-infection (87.5–93.3%) but had slightly lower infection rates than those of *C. jejuni* by 7 days post-infection (66.7–75.0 vs. 72.2–85.7%, respectively). By contrast, *C. coli* had the lowest infection rates among the three strains.

**FIGURE 2:**
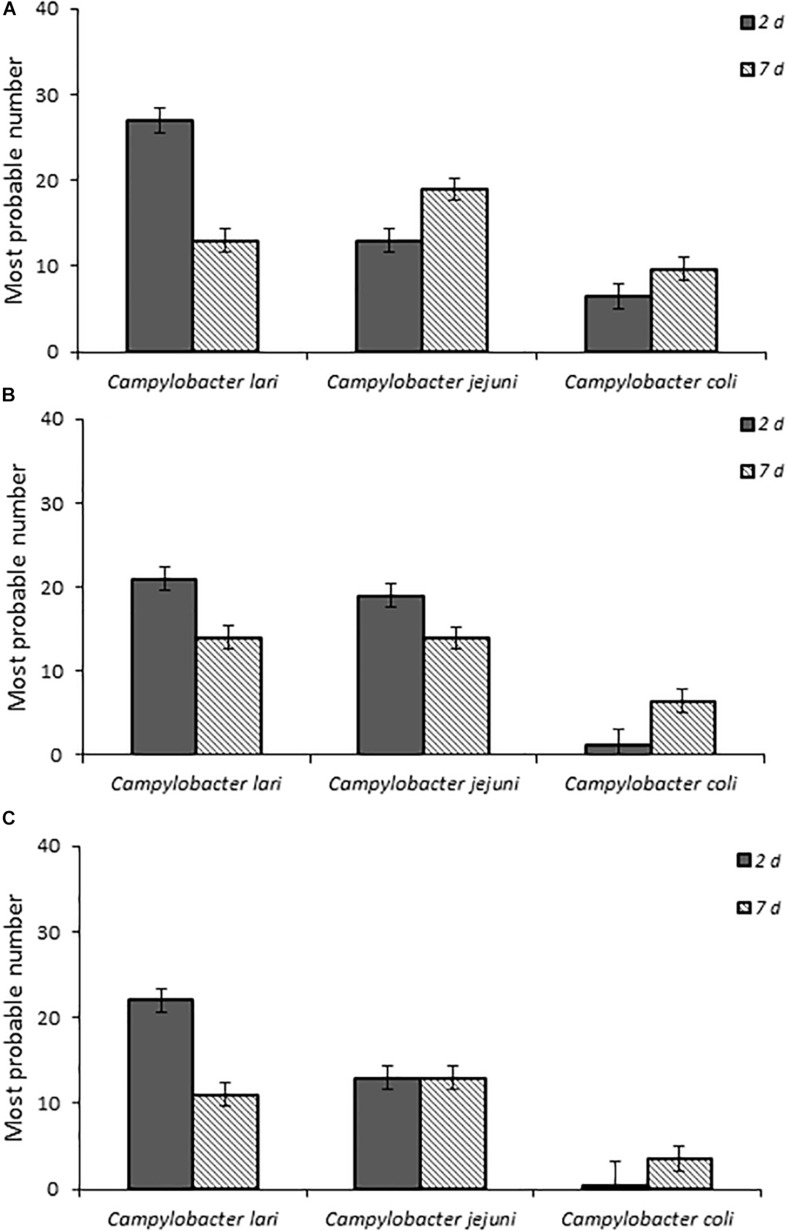
Fecal bacterial burden in infected chicks with *Campylobacter* spp. laboratory isolates at doses **(A–C)**, respectively, as determined by the most probable number (MPN) calculations as described in section “Materials and Methods” and in Supplementary Table S1.

When fecal bacterial burden following infection was examined by using MPN, the chicks infected with *C. lari* had the highest detectable bacterial load followed by those infected by *C. jejuni* and *C. coli* for each dose at 2 days post-infection (Figure 2). Both *C. jejuni* and *C. lari* infections were more persistent than those of *C. coli*. We found that *C. jejuni* easily infected 1-day-old chicks even at the lowest inoculum dose and had the most consistent rate and persistence of infection among the laboratory strains. When inoculated with the same dose as *C. jejuni*, we found that *C. coli* infected fewer chicks at the same inoculum dosages (Supplementary Table S1). No overt disease symptoms were observed in the chicks infected by the three *Campylobacter* species. The natural ingestion exposure route developed in our study enabled successful infection of the young chicks with both the laboratory and clinical strains of *Campylobacter (C. jejuni, C. lari*, and *C. coli*) with minimal physiological distress.

### Infection of Chicks With *Campylobacter* Clinical Isolates

**FIGURE 3:**
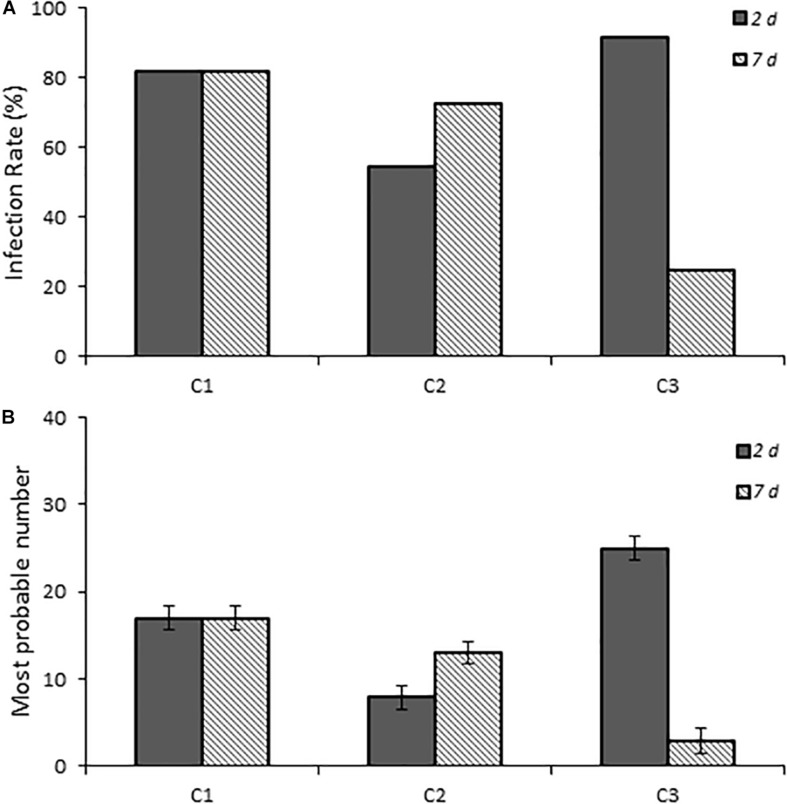
Infection rates and fecal burden levels of *Campylobacter* from chicks infected with clinical isolates of *Campylobacter* spp. **(A)** shows chicks infected with dose B *Campylobacter* spp. Percent infected was determined by dividing the number of infected chicks by the total number of chicks inoculated with the various *Campylobacter* species. Chicks were considered infected if fecal samples collected at the various times were positive for *Campylobacter* spp. as determined by using the passive-filter plating as described in the section “Materials and Methods.” **(B)** shows the fecal burden as measured by using the most probable number calculator.

One-day-old chicks were inoculated with three *C. jejuni* isolates collected from patients with campylobacteriosis to determine if human *Campylobacter* isolates are also capable of establishing infection in this chick model of infection. All three isolates successfully established infection and persisted to 7 days post-infection (Figure 3). Although C1 showed a consistent infection rate (81.8%) at both time points, C2 and C3 were more variable. C2 had a lower infection rate at 2 days post-infection (54.4%, *p* ≤ 0.019) than at 7 days post-infection (72.7%), whereas C3 had the reverse trend (91.7 and 25.0%, respectively).

### Infection of Environmental *Campylobacter* spp. Isolates

Four California gull isolates (58BB, 63A, 64BB, and 70BB), which were homologous to *C. lari* (≥94% homology), *C. jejuni* (≥97% homology), *C. lari* (≥97% homology), and *C. volucri* (≥97% homology), respectively, were capable of infecting chicks via this chick model. *C. volucri*, a new *Campylobacter* species isolated from black-headed gulls (*Larus ridibundus*) (Debruyne et al., 2010) is more closely related to *C. jejuni* than to *C. lari* according to phylogenetic analysis of 16S rRNA and hsp60 gene sequences (Debruyne et al., 2010).

**FIGURE 4:**
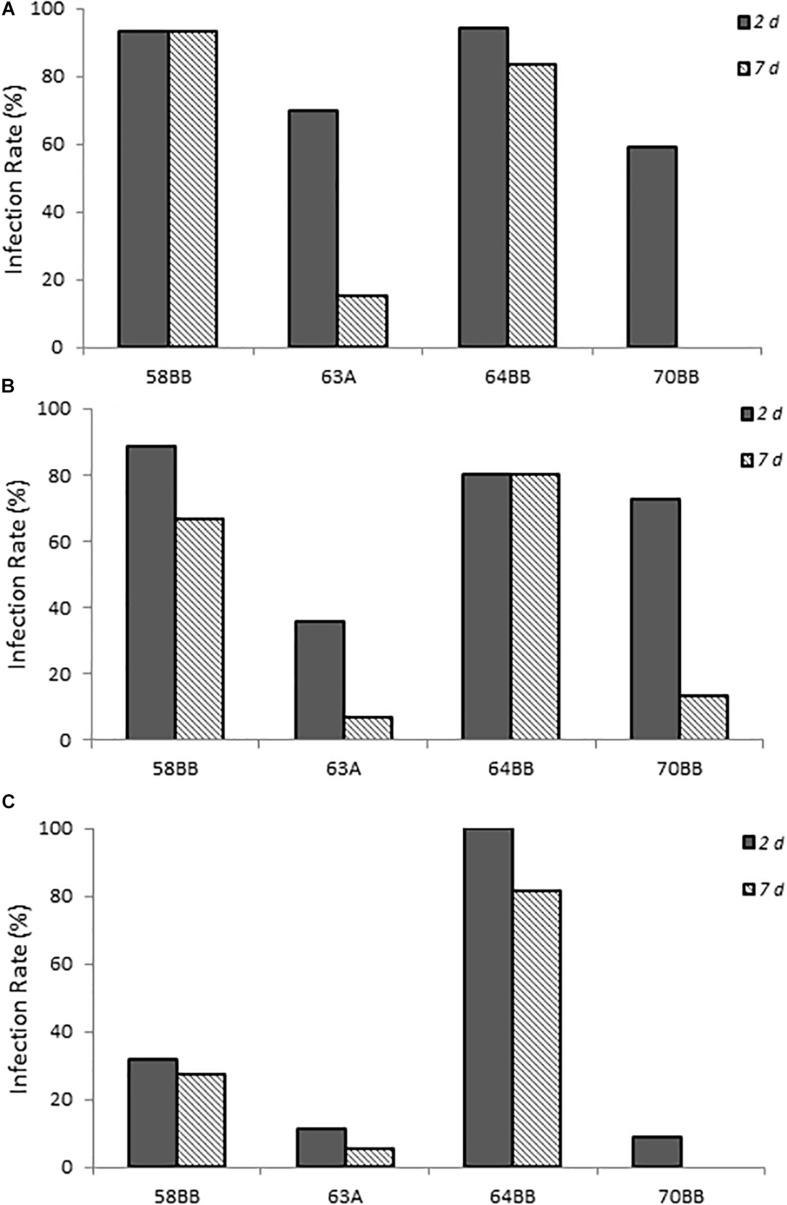
Infection rates of environmental isolates of *Campylobacter* spp. **(A–C)** show chicks infected with *Campylobacter* spp. environmental isolates at doses A–C, respectively, as described in Supplementary Table S1. Percent infected was determined by dividing the number of infected chicks by the total number of chicks inoculated with the various *Campylobacter* species. Chicks were considered infected if fecal samples collected at the various times were positive for *Campylobacter* spp. as determined by using the passive-filter plating as described in section “Materials and Methods.”

The two *C. lari*-like isolates (58BB and 64BB) showed the highest infection rates at the two highest doses, with >80% of the animals infected at 2 days post-infection and the infection persisting to 7 days post-infection (Figure 4). Even at the lowest dose, 64BB continued this trend, with >80% of the animals infected on both days. At the lowest dose, 58BB was less infective and infected <32% of the animals on each day. By contrast, chicks inoculated with 70BB *C. volucri-*like or 63A *C. jejuni*-like isolates had consistently lower rates of infection than those of both *C. lari*-like isolates, with more variations in their infection rates. The *C. volucri*-like isolate had a high infection rate of 72.7% on 2 days post-infection for dose B, which dropped to 13.6% by 7 days post-infection. Similarly, the *C. jejuni*-like isolate had a high infection rate of 70.0% at 2 days post-infection for dose A, which dropped to 15.0% at 7 days post-infection (Figure 4).

**FIGURE 5:**
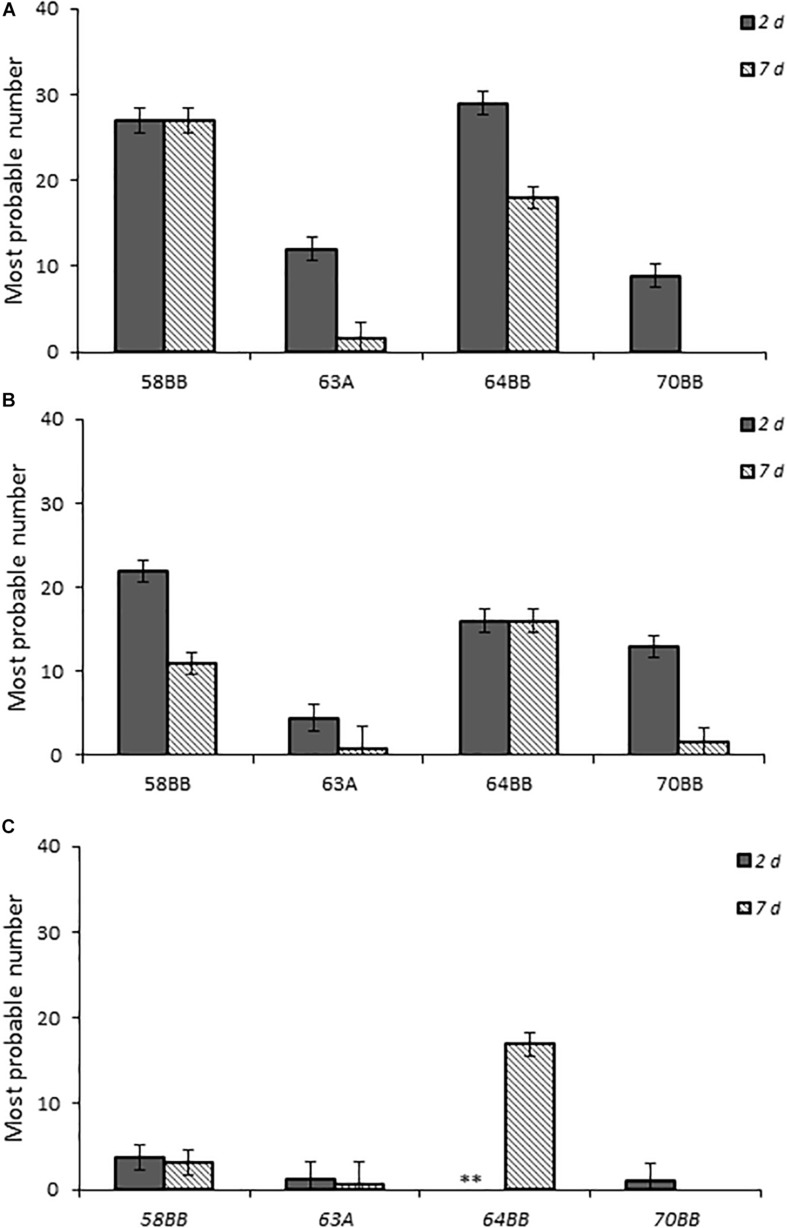
Fecal bacterial burden in chicks infected with *Campylobacter jejuni*. Environmental isolates at doses **(A–C)**, respectively, as determined by the most probable number (MPN) calculations as described in section “Materials and Methods.”

Chicks that received the highest inoculum dose of 64BB and 58BB also had the highest *Campylobacter* burden, as indicated by MPN, compared with those of 63A and 70BB at both time points tested (Figure 5). The overall *Campylobacter* burden observed from infection with 63A and 70BB, although similar to each other, was lower than the bacterial fecal burdens from animals infected with the 64BB and 58BB isolates (*p* ≤ 0.009).

## Discussion

The *G. gallus* chick species has long been used as an important model for investigation of bacterial colonization factors, especially for *Campylobacter* infection, because of their preferential colonization of avian guts under optimal growth conditions (Wassenaar et al., 1993; Cawthraw et al., 1996; Hendrixson and DiRita, 2004; Stern, 2008). In the previous studies, bacterial strains, including laboratory-adapted *C. jejuni* isolates and *C. jejuni* isolates from chicken, patients, and waterborne outbreaks, were administered via oral gavage to chickens of various ages, tissue or cecal samples were collected, and samples were enumerated by plating of serial dilutions of samples. In those experiments, the cecum was found to be the main site of colonization, although organisms were also recovered throughout the gastrointestinal tract as well as the spleen and liver. Unlike the previous chick models of infection, the model in our studies was one we developed to be less invasive than others by infecting animals with *Campylobacter* through their drinking water, which mimics the natural route of infection, uses a sampling procedure that measures bacterial burden from freshly excreted fecal samples, and uses a passive filtration *in vitro* culture procedure as a more feasible model to assess *Campylobacter* infectivity. Especially, the developed model was further used to evaluate the infectivities of different *Campylobacter* species and *Campylobacter* isolates from wild fowl, which have not been documented previously.

This study also demonstrated successful infection and persistence (≤21 days post-infection) (data not shown) of *Campylobacter* spp. from three different groups (laboratory-maintained isolates, clinical isolates from human specimens, and environmental isolates from fresh fecal samples of California gull) in newly hatched chicks. This approach also provided the sensitivity to reveal variations of *Campylobacter* infectivity (e.g., virulence) among the different species, isolates, and environmental genotypes evaluated. For example, the laboratory isolates (*C. lari* and *C. jujuni)*, which had been maintained under *in vitro* laboratory growth conditions for an unknown extended period of time, resulted in the highest infection rates and fecal bacterial burdens, whereas *C. coli* resulted in the lowest (Figures 1, 2). More importantly, the results from the California gull isolates revealed that environmental isolates released from avian reservoirs can infect chicks. Among the gull isolates, 64BB (*C. lari-*like genotype) exhibited infectivity and persistence rates comparable to those of the *C. jejuni* clinical isolate. Isolate 58BB (*C. lari-*like genotype) exhibited infectivity and persistence rates comparable to those of the *C. lari* laboratory isolate (Figures 4, 5). The observed differences in infectivity between the environmental, clinical, and laboratory isolates could be attributed to differential expression of bacterial virulence factors and/or host immune responses during infection.

Furthermore, this model may help us to understand the bacterial burden and its release through defecation. Previously, it has been shown that 1-day-old chicks, orally challenged with a 10^4^CFU *C. jejuni* isolate or as few as 30 CFUs, which experienced a single passage of model chicks, achieved maximal cecal colonization within 3 days at levels of ≤1 × 10^10^CFU g^–1^ cecal contents (Cawthraw et al., 1996). In the other studies for challenge of 2-day-old chicks, *C*. *jejuni* has been observed at up to ∼10^8^ CFU g^–1^ of cecal contents (Wassenaar et al., 1993; Cawthraw et al., 1996). Once *Campylobacter* levels are established, they tend to remain at high levels throughout the life of the chick (Stern, 2008). In this study, a high bacterial cecal burden was stable at 10^6^ to 10^8^ CFU g^–1^ of cecal material throughout the duration of this experiment. The majority (57%)of positive samples from all isolates contained ≥7.5 × 10^4^ CFU g^–1^ of feces. Assuming an average release of 1.5 g of fecal material for each defecation, 29.94 g of total solids per hen-day, and voids 20 times a day (Huttly et al., 1998), each individual host is capable of releasing >3.0 × 10^5^ culturable bacteria into the environment with each defecation event or 6.0 × 10^6^ culturable bacteria into the environment per day.

In conclusion, this new method provides not only the ability to monitor infection through time course but also to assay virulence and other pathogen factors with relative ease. Although the relationship between the colonization in the chick ceca and the mammalian gut is unknown, the chick model still provides a relevant and natural host for *Campylobacter* infection. This new approach can also lend itself to identifying novel *Campylobacter* virulence factors, understanding host immune responses following infection with *Campylobacter*, screening for potential therapeutic agents, and developing vaccines relevant to the poultry industry and human health (Nedrud, 1999; Nassar, 2018). Lastly, the levels and persistence of infectious bacteria released in feces within the chick gastrointestinal tract monitored over time are important parameters when assessing the importance of waterfowl in transmission and exposure to zoonotic *Campylobacter* spp. in beach sites used for human recreation.

## Data Availability Statement

The datasets generated for this study are available on request to the corresponding author.

## Ethics Statement

The animal study was reviewed and approved by Institutional Animal Care and Use Committee (IACUC). Written informed consent was obtained from the owners for the participation of their animals in this study.

## Author Contributions

DL and JL designed and supervised all experiments, did some data analysis, performed some experiments, and prepared the manuscript. IS did qPCR and summarized qPCR data. TG did some data analysis. KO helped to initiate the experiments and made a critical review. EV coordinated the experiments, meeting, and data analysis, and helped some manuscript preparation.

## Conflict of Interest

IS was employed by Pegasus Technical Services, Inc. The remaining authors declare that the research was conducted in the absence of any commercial or financial relationships that could be construed as a potential conflict of interest.
