# Supplementary material for: A Gallus gallus Model for Determining Infectivity of Zoonotic Campylobacter
Source: Front Microbiol. 2019 Oct 22;10:2292. doi: 10.3389/fmicb.2019.02292 (PMC6817472; doi:10.3389/fmicb.2019.02292)
Supplement: Supplementary file 1 [file Table_1.docx]

Table S1. Total ingested bacteria (CFU)

|  | Clinical Isolates | | | Environmental Isolates | | | | Laboratory Isolates | | |
| --- | --- | --- | --- | --- | --- | --- | --- | --- | --- | --- |
| Dose | C1 | C2 | C3 | 58BB | 63A | 64BB | 70BB | *C. lari* | *C. jejuni* | *C. coli* |
| A | ND | ND | ND | 1.0–3.7 × 10^7^ | 1.0–9.0 × 10^7^ | 1.1–6.6 × 10^7^ | 2.4–6.0 × 10^7^ | 1.1–3.0 × 10^7^ | 1.1–7.5 × 10^7^ | 1.2–7.5 × 10^7^ |
| B | 2.8–5.0 × 10^6^ | 1.1–2.7 × 10^6^ | 1.0–9.0 × 10^6^ | 1.0–9.6 × 10^6^ | 1.0–9.5 × 10^6^ | 2.4–7.8 × 10^6^ | 1.8–7.5 × 10^6^ | 1.0–8.0 × 10^6^ | 1.1–6.6 × 10^6^ | 1.0–6.0 × 10^6^ |
| C | ND | ND | ND | 1.1–4.8 × 10^5^ | 4.0–9.0 × 10^5^ | 2.1–9.3 × 10^5^ | 2.7–9.0 × 10^5^ | 1.1–9.0 × 10^5^ | 2.1–8.4 × 10^5^ | 3.6–9.0 × 10^5^ |

CFU: colony-forming unit

ND: not done.

*C.*: *Campylobacter*

Table S2. Differential biochemical and phenotypic characteristics of *Campylobacter* isolates from gull excreta in California by multilocus sequence typing (MLST)

| Isolate | *aspA* type | *atpA* type | *glnA* | *gltA* | *pgmA* | *tktA* | Sum |
| --- | --- | --- | --- | --- | --- | --- | --- |
| 58BB | 88% similar to  *C. lari,* CP007771 | 94% similar to  *C. lari*, CP007778 | 95% similar to  *C. lari*, CP007776 | - | - | 94% similar to  *C. lari,* CP007777 | *C. lari* like |
| 63A | 99% similar to  *C. jejuni,* CP022080 | 99% similar to  *C. jejuni,* CP023343 | 99% similar to  *C. jejuni,* CP023343 | 99% similar to  *C. jejuni,* CP023343 | 99% similar to  C. jejuni, CP022076 | 97% similar to  *C. jejuni*, CP002029 | *C. jejuni* |
| 64BB | - | - | - | 97% similar to  *C. lari,* CP000932 | 97% similar to  *C. lari*, CP000932 | 98% similar to  *C. lari*, CP007778 | *C. lari* like |
| 70BB | 98% similar to  *C. volucri,* CP007774 | 99% similar to  *C. volucri,* CP007774 | 99% similar to  *C. volucri,* CP007774 | 99% similar to *C. volucri*, CP007774 | 99% similar to *C. volucri*, CP007774 | 97% similar to *C. volucri*, CP007774 | *C. volucri* |

Table S3. Physiological and biochemical characteristics of *Campylobacter* 63A

| Pattern no. or species | 58BB | 112BB | 70BB | 5A | 64BB | 63A | *C. jejuni* | *C. coli* | *C. lari* |
| --- | --- | --- | --- | --- | --- | --- | --- | --- | --- |
| Hemolysis | γ | α | γ | γ | γ | γ | γ | γ | γ |
| Urease | - | - | - | - | - | - | - | - | - |
| Oxidase | - | - | + | + | + | + | + | + | + |
| Catalase | + | + | + | + | + | + | + | + | + |
| Growth on  MacConkey Agar | + | - | - | - | + | + | + | + | + |
| Growth on Nutrient Agar | - | + | - | - | - | - | + | +, but faint | +, but faint |
| Growth at 30˚C (after 72 h) | - | + | + | - | + | + | + | + | + |
| Hippurate Hydrolysis | - | - | - | - | - | + | + | - | - |
| Growth in 1% glycine | + | + | - | - | + | + | + | + | + |
| Growth in 3.5% NaCl | - | + | - | - | - | - | - | - | - |
| Growth at 42˚C (after 72 h) | + | + | + | + | + | + | + | + | + |
| Nitrate reduction | + | + | + | + | + | + | + | - | + |
| Growth at 37˚C (after 72 h) | + | + | + | + | + | + | + | + | + |
| Anaerobic growth at 37˚C | + | + | + | + | + | + | + | + | + |
| Nitrite Reduction | - | - | - | - | - | - | - | - | - |
| Resistance to Cephalothin | Resistant | Susceptible | Susceptible | Susceptible | Resistant | Resistant | Resistant | Resistant | Resistant |
| Production on TSI Agar of H2S | - | - | - | - | - | - | - | + | + |
| Production of acid on TSI Agar | - | + | - | - | - | - | - | - | - |
| Growth at 25˚C (after 72 h) | - | + | - | - | - | - | - | - | - |
| Growth in ambient O2 | - | + | - | - | - | - | - | - | - |
| Colony Morphology | individual colonies, no swarming | Heavy growth, but no swarming evident | individual colonies, half or less swarming | individual colonies, seldom swarming | no swarming evident | swarming and non |  |  |  |
| Nalidixic Acid Resistance | Susceptible | Partially susceptible (zone around disc only partially cleared; unable to accurately measure size) | Resistant | Resistant | Resistant | Susceptible | Susceptible | Susceptible | Resistant |

Table S4. Oligonucleotide PCR primer sequences for *Campylobacter* genus and species-specific genes along with amplified PCR fragment sizes

| Species | Genes | Sequences (5'-3') | Reference | PCR products (bp) |
| --- | --- | --- | --- | --- |
| *C. jejuni* | *mapA* | GGA CGG TAA CTA GTT TAG TAT T | Stucki et al. (1995) | 589 |
|  |  | CTA TTT TAT TTT TGA GTG CTT GTG |  |  |
| *C. coli* | *ceuE* | AAT TGA AAA TTG CTC CAA CTA TG | Gonzalez et al. (1997) | 462 |
|  |  | TGA TTT TAT TAT TTG TAG CAG CG |  |  |
| *C. lari* | 16S–23 S rRNA  ITS region | CTT ACT TTA GGT TTT AAG ACC | Khan and Edge (2007) | 279 |
|  |  | CAA TAA AAC CTT ACT ATC TC |  |  |
| *Campylobacter* spp. | 16S rRNA | CACGTGCTACAATGGCATAT | Lund et al. (2004) | qPCR |
|  |  | GGCTTCATGCTCTCGAGTT |  |  |
|  |  | FAM-CAGAGAACAATCCGAACTGGGACA-TAMRA |  |  |
